# Ponce De Leon Spring

**Published:** 1873-06

**Authors:** 


### PART III.

### EDITORIAL AND MISCELLANEOUS.

*Ponce de Leon Spring.*

The public for some time past has been familiar with the name and the great medicinal virtues of this spring. No doubt in some particulars its waters have been credited with healing properties in a strain of extravagant comment; still, its really curative elements have, in the main, been only partially made apparent to the general public.

Although as yet no strict analysis of the ingredients has been made, it has within our personal knowledge effected some very remarkable cures, especially affections of the kidneys, of the bladder, and also some wonderful cases of dyspepsia. Nature seems to have gathered here, as in the healing pool of Siloam, some of the rarest and most wonderful remedies for the above-mentioned diseases, which so commonly afflict mankind. This dispensatory of an all-wise Providence opens its treasures to the suffering all the year round, and is a permanent blessing.

Knowing these facts, we deem it our duty to give as much publicity to these waters as possible by means of our journal, in order that all who may be afflicted by such diseases can learn of this beneficent provision of Nature, and, if their physicians so advise, partake of its benefits.

A very pleasant and comfortable hotel has been recently erected in a beautiful grove adjacent to the spring for the accommodation of visitors. Those who may desire to reside in the city can ride out to the hotel and spring at any hour of the day, as the Ponce de Leon spring is situated only about one and a half miles from the center of the city. For further particulars in any respect, address Messrs. Bell & Goldsmith, Atlanta, Georgia.

